# Relationships between glucose, energy intake and dietary composition in obese adults with type 2 diabetes receiving the cannabinoid 1 (CB1) receptor antagonist, rimonabant

**DOI:** 10.1186/1475-2891-11-50

**Published:** 2012-07-23

**Authors:** Charlotte Heppenstall, Susan Bunce, Jamie C Smith

**Affiliations:** 1 Department of Diabetes & Endocrinology, Torbay Hospital, Lawes Bridge, Torquay, Devon, TQ2 7AA, UK

**Keywords:** Dietary assessment, Dietary intervention, Drug interventions, Diabetes

## Abstract

**Background:**

Weight loss is often difficult to achieve in individuals with type 2 diabetes and anti-obesity drugs are often advocated to support dietary intervention. Despite the extensive use of centrally acting anti-obesity drugs, there is little evidence of how they affect dietary composition. We investigated changes in energy intake and dietary composition of macro- and micronutrients following therapy with the endocannabinoid receptor blocker, rimonabant.

**Methods:**

20 obese patients with type 2 diabetes were studied before and after 6 months dietary intervention with rimonabant. Dietary intervention was supervised by a diabetes dietician. Five-day food diaries were completed at baseline and at 6 months and dietary analysis was performed using computer software (Dietplan 6).

**Results:**

After 6 months, (compared with baseline) there were reductions in weight (107 ± 21Kg versus 112 ± 21, p < 0.001, 4% body weight reduction), and improvements in HbA1c (7.4 ± 1.7 versus 8.0 ± 1.6%, p < 0.05) and HDL cholesterol. Intake of energy (1589 ± 384 versus 2225 ± 1109 kcal, p < 0.01), carbohydrate (199 ± 74 versus 273 ± 194 g, p < 0.05), protein (78 ± 23 versus 98 ± 36 g, p < 0.05), fats (55 ± 18 versus 84 ± 39 g, p < 0.01) and several micronutrients were reduced. However, relative macronutrient composition of the diet was unchanged. Improvement in blood glucose was strongly correlated with a reduction in carbohydrate intake (r = 0.76, p < 0.001).

**Conclusions:**

In obese patients with type 2 diabetes, rimonabant in combination with dietary intervention led to reduced intake of energy and most macronutrients. Despite this, macronutrient composition of the diet was unaltered. These dietary changes (especially carbohydrate restriction) were associated with weight loss and favourable metabolic effects.

## Introduction

Obesity is extremely common in type 2 diabetes and is a major contributor to premature morbidity and mortality [1,2]. Obesity results from an imbalance of energy intake and energy expenditure and so any strategy to reduce body weight must rely on either, a reduction in energy intake, an increase in energy expenditure or both. Supervised weight-loss through dietary intervention is therefore considered a cornerstone in the management of obese individuals with type 2 diabetes [3]. However, most strategies used to combat obesity have not yielded long-term success and so there is increasing interest in the use and development of pharmacological agents to tackle obesity [4]. Centrally-acting anti-obesity drugs such as sibutramine or the CB1 receptor antagonist rimonabant are considered to act principally by reducing appetite and/or increasing satiety, thereby producing reduced energy intake [4,5]. For example, in the RIO-trial programme involving obese subjects with type 2 diabetes and other cardiovascular risk factors, rimonabant in combination with a recommended moderate daily caloric reduction of 600 kcal was extensively studied in terms of weight, metabolic and cardiovascular parameters [6]. However, despite these large clinical trials and widespread clinical use there is little evidence of how centrally acting anti-obesity drugs specifically affect dietary composition in humans.

In the case of rimonabant, animal data suggest that weight loss occurs not only because of reduced energy intake, but also due to increased energy expenditure through increased fat oxidation in adipose tissue [7]. In addition, animal data also suggest that CB1 receptor antagonism in the rat hypothalamus leads to a preferential reduction in the intake of palatable fatty and sugary food [8,9]. To date, these findings have not been demonstrated in human studies. Because of concerns relating to depression and suicidal risk, rimonabant’s license was withdrawn by the European regulatory authorities in 2008 but there is still interest in this therapeutic class with other agents in development [5].

In recent times there has been substantial interest in the way macronutrient intake affects both weight and glycemia in type 2 diabetes [3], especially the effects of restricting carbohydrate intake [10-12]. Several studies have reported benefits in terms of improved glucose control when a low carbohydrate diet is compared with conventional intake of carbohydrate [10,13-15] although evidence is conflicting with not all studies demonstrating these benefits [12,16].

The aim of this study was as follows:- [1] to assess weight and metabolic changes following rimonabant therapy in obese patients with type 2 diabetes; [2] to investigate in detail, changes in energy, macro- and micro-nutrient intake following rimonabant therapy; and [3] to investigate how changes in macronutrient intake (eg. carbohydrate, fat) might influence changes in weight and glycaemia.

## Methods

### Study design and subjects

Twenty subjects (age range 30-70 yrs) (11 male, 9 females) with type 2 diabetes (11 insulin-treated) were recruited from the multidisciplinary diabetes clinic at Torbay Hospital, a UK district general hospital in 2008. All subjects were obese with a body mass index of greater than 30 Kg/m^2^ and had expressed the desire to lose weight. In an open design, all subjects were studied before, during and after 6 months dietary and lifestyle intervention and rimonabant therapy, 20 milligrams once daily (the standard licensed dose).

At baseline, all subjects received dietary and lifestyle advice from a specialist diabetes dietician, having completed a 5 day food diary, and were prescribed an individualised 600-800 kcal deficit diet based on healthy eating and portion control. Patients were followed-up during the trial with monthly telephone consultations and with outpatient clinic reviews at 3 and 6 months. Patients with contraindications for the use of rimonabant (including depressive illness), clinical or echocardiographic evidence of left ventricular impairment, peripheral vascular disease or significant renal impairment (estimated GFR < 30 ml/min) were excluded. All subjects had been receiving a stable regime of blood glucose-lowering treatment for at least 3 months prior to study entry. All subjects were studied at baseline and then at 6 months following the weight loss intervention. Study measurements and blood samples were undertaken in the morning after a 12 hour fast and medications were omitted on the morning of study. Dietary follow up consisted of monthly telephone calls and review and analysis of the food diary at 6 months. The telephone calls involved informal discussion with the individual patient about general well-being, tolerability of the study medication, appetite and food intake. Patients were given encouragement to adhere to their individualised diet plan. The 5 day food diaries at baseline and at 6 months were analysed using the dietary analysis software package, Diet Plan 6 (Forestfield Software Ltd, UK). The 5 day food diaries were discussed with the individual patients during the baseline and 6 monthly visits. A food portion Atlas [17] was used with the patient so that the patient could identify their portion size. This was coded by the dietitian and the amounts entered into the Diet Plan programme.

Body composition analysis was performed using the method of bioelectrical impedance (Tanita).

Fasting blood samples (10 ml venous blood for each subject) were drawn on the morning of study. The study had approval from the local research ethics committee, together with clinical trial authorisation. All subjects gave informed consent to participate in the study.

### Statistical analysis

All statistical analyses were performed using SPSS (version 14) for Windows. Data are expressed as mean values ± SD. Paired t-tests were used to evaluate differences between group means in the same subjects over time for normally distributed data. Correlation between variables was evaluated using Spearman’s and Pearson’s correlation coefficients. A p value of less than 0.05 was considered significant.

## Results

The study group consisted of 11 males and 9 females. The mean age of participants was 58 ± 11 years and the mean duration of diabetes was 7 ± 7 years. Eleven out of 20 were receiving insulin therapy. Mean Body mass index (Kg/m^2^) was 38 ± 5 Kg/m^2^. There was 1 active smoker in the group.

The effects of rimonabant on physical and biochemical measurements are shown in Table 1. Weight was reduced from 112 ± 21 Kg at baseline to 107 ± 21 Kg after 6 months (p < 0.001) equating to a mean 4% weight reduction and this was accompanied by a reduction in waist circumference from 124 ± 13 cm to 121 ± 13 cm (p < 0.05). Nine out of 20 patients lost more than 5% of body weight over the 6 month treatment period and 2 out of 20 lost more than 10% of body weight. Weight changes were associated with a reduction in HbA1c from 8.0 ± 1.6% at baseline to 7.4 ± 1.7% at 6 months (p < 0.05). For the 11 subjects receiving insulin therapy, mean insulin dose fell from 116 ± 59 units/day at baseline to 102 ± 71 units/day after 6 months (p < 0.05). There was a rise in HDL cholesterol from 1.2 ± 0.2 mmol/L at baseline to 1.3 ± 0.2 mmol/L at 6 months (p < 0.01).

**Table 1:** Effects of Rimonabant with dietary intervention on physical and biochemical parameters over the study period (n = 20) (Mean ± SD)

	**Baseline**	**6 Months**
Weight (Kg)	112 ± 21	107 ± 21***
Waist circumference (cm)	124 ± 13	121 ± 13**
Fat mass (Kg)	44 ± 12	42 ± 12
HbA1c (%)	8.0 ± 1.6	7.4 ± 1.7*
Total cholesterol (mmol/L)	4.4 ± 1.0	4.4 ± 1.0
LDL cholesterol (mmol/L)	2.3 ± 0.8	2.4 ± 0.9
HDL cholesterol (mmol/L)	1.2 ± 0.2	1.3 ± 0.2**
Triglyceride (mmol/L)	1.9 ± 1.1	1.7 ± 0.9
Insulin dose (units / day)	116 ± 59	102 ± 71*
Systolic blood pressure (mmHg)	147 ± 21	142 ± 24
Diastolic blood pressure (mmHg)	85 ± 8	83 ± 11

*p < 0.05 in comparison to baseline.

**p < 0.01 in comparison to baseline.

***p < 0.001 in comparison to baseline.

### Macronutrient intake and dietary composition

Completed and detailed dietary data using Dietplan was available for 18 out of 20 subjects. Food diaries were deemed incomplete and not suitable for analysis in 2 subjects. The changes in daily dietary intake of energy and macronutrients during the study are shown in Table 2. Six months following rimonabant treatment daily energy intake was reduced from 2225 ± 1109 to 1589 ± 384 kcal (p < 0.01). Total carbohydrate intake was reduced from 273 ± 194 to 199 ± 74 g (p < 0.05) with statistically significant reductions in starch (148 ± 48 versus 114 ± 36 g, p < 0.05) and non-starch polysaccharide (18 ± 5 versus 14 ± 3 g, p < 0.01) but a non-significant reduction in intake of sugar (121 ± 171 versus 74 ± 64 g (p = 0.10). Protein intake was reduced from 98 ± 36 to 78 ± 23 g (p < 0.05). Total fat intake was reduced from 84 ± 39 to 55 ± 18 g (p < 0.01) (35% reduction) with reductions in saturated fatty acids (30 ± 16 versus 20 ± 6 g, p < 0.01) (33% reduction), monounsaturated fatty acids (29 ± 12 versus 19 ± 7 g, p < 0.01) (34% reduction) and polyunsaturated fatty acids (16 ± 8 versus 10 ± 4 g, p < 0.01) (38% reduction).

**Table 2:** Effects of Rimonabant with dietary intervention on daily energy and macronutrient intake before and after intervention (n = 18) (Mean ± SD)

**Variable**	**Baseline**	**6 months**
Energy (Kcal)	2225 ± 1109	1589 ± 384 **
Carbohydrate (g)	273 ± 194	199 ± 74 *
Protein (g)	98 ± 36	78 ± 23 *
Fat (g)	84 ± 39	55 ± 18 **
Starch (g)	148 ± 48	114 ± 36 *
Sugar (g)	121 ± 171	74 ± 64
Non-starch polysaccharide (g)	18 ± 5	14 ± 3 **
Saturated fatty acids (g)	30 ± 16	20 ± 6 **
Monounsaturated fatty acids (g)	29 ± 12	19 ± 7 **
Polyunsaturated fatty acids (g)	16 ± 8	10 ± 4 **

*p < 0.05 in comparison to baseline.

**p < 0.01 in comparison to baseline.

The dietary composition of macronutrients for the group, expressed as a % of total energy intake at baseline was carbohydrate 46%, fat 34% and protein 17%. After 6 months this composition had not changed significantly with carbohydrate 47%, fat 31% and protein 18%.

### Micronutrient intake

Details of daily micronutrient intake before and after rimonabant treatment are shown in Table 3. With respect to micronutrient intake, after 6 months (in comparison with baseline), levels of intake of sodium (2544 ± 866 versus 3793 ± 1487 mg, p < 0.01), potassium (2874 ± 944 versus 4307 ± 3299 mg, p < 0.01), vitamin E (5.9 ± 2.6 versus 8.8 ± 5.1 mg, p < 0.05) and folate (292 ± 103 versus 431 ± 324 mg, p < 0.05) were reduced. There was no evidence of a statistically significant increased intake of any micronutrient studied following the intervention. In terms of Reference Nutrient Intake (RNI) for micronutrients, 9 of the 20 subjects did not meet the RNI for potassium at baseline and this increased to 15 at 6 months. No subjects over the age of 65 met the RNI for Vitamin D. With regards to calcium intake, 7 of the 20 subjects did not meet the RNI at baseline and 9 did not meet the RNI at 6 months. With regards to iron intake 3 subjects did not meet the RNI at baseline and this increased to 5 at 6 months. For vitamin A, 4 subjects did not meet the RNI at baseline and this increased to 5 at 6 months.

**Table 3:** Effects of Rimonabant with dietary intervention on daily micronutrient intake before and after intervention (n = 18) (Mean ± SD)

**Variable**	**Baseline**	**6 months**	**Reference Nutrient Intakes (RNI)**
Calcium (mg)	940 ± 439	725 ± 272	700
Iron (mg)	14 ± 5	11 ± 4	8.7
			Females < 50 yrs 14.8
Vitamin E (mg)	8.8 ± 5	5.9 ± 3 *	No RNI
Folate (μg)	431 ± 324	292 ± 103 *	200
Vitamin A (μg)	3075 ± 3610	3688 ± 2955	Males 700
			Females 600
Vitamin C (mg)	299 ± 704	135 ± 121	40
Sodium (mg)	3793 ± 1487	2544 ± 866 **	1600-2400
Potassium (mg)	4307 ± 3299	2874 ± 944 *	3500

*p < 0.05 in comparison to baseline.

**p < 0.01 in comparison to baseline.

### Relationship between glycaemia and dietary composition

**Figure 1:**
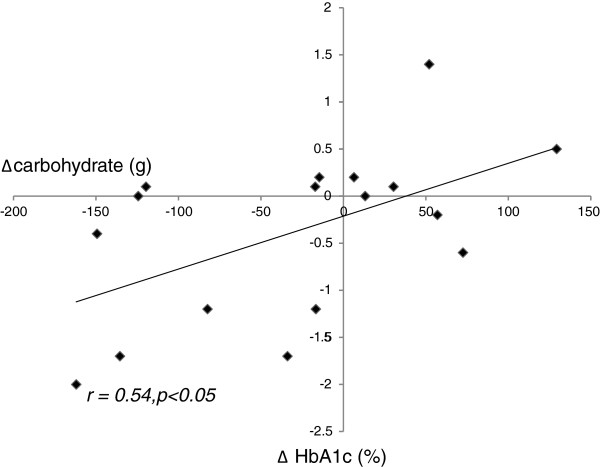
Relationship between reduction in carbohydrate intake and improvement in glycaemic control (as indicated by change in HbA1c concentration) in rimonabant-treated subjects (n = 17) (r = 0.54, p < 0.05).

A strong correlation was observed between the reduction in carbohydrate intake and improvement in glycaemic control (as indicated by change in HbA1c concentration) over the study period (r = 0.76, p < 0.0001). This correlation persisted after controlling for weight loss and energy intake. Multiple stepwise regression analysis revealed carbohydrate intake to be an independent predictor of change in HbA1c (Table 4). It was noted that 1 individual recorded an excessively large carbohydrate intake at baseline, which reduced dramatically following the intervention. This led to a very high change in carbohydrate intake for this individual of around 550 g. On further enquiry, the carbohydrate intake was verified. Results were re-analysed excluding this individual but the statistical relationship persisted (r = 0.54, p < 0.05) (Figure 1). Reduction in fat intake correlated with degree of weight loss (change in weight from baseline to end of study) (r = 0.48, p < 0.05). In contrast, reduction in carbohydrate intake did not correlate with the degree of weight loss.

**Table 4:** **Stepwise multiple regression analysis with ΔHbA1c as dependent variable (R**^**2**^ **= 0.58)**

**Independent variable**	**Beta**	**t**	**p value**
Δ carbohydrate intake	0.76	4.7	<0.0001
Δ energy intake	0.16	0.61	0.16
Δ weight	-0.26	-0.15	0.88

## Discussion

Dietary intervention is fundamental to the control of glucose and weight in obese individuals with type 2 diabetes. Understanding how specific dietary interventions and anti-obesity agents affect an individual’s dietary composition and energy intake should enable us to target these interventions more effectively. In the present study we investigated the effects of a dietary intervention involving the use of the anti-obesity agent, rimonabant on blood glucose, energy intake and dietary composition in obese adults with type 2 diabetes.

The main findings of the present study were that an individualised diet based on healthy eating and portion control, in combination with rimonabant therapy led to significant weight loss over a 6 month period with a majority of patients losing in excess of 5% of their body weight. The reduction in body weight was accompanied by a reduction in weight circumference and favourable changes to the lipid profile and blood glucose control. These beneficial effects on body weight and metabolic parameters occurred in association with a significant reduction in energy intake, together with reductions in the intake of the principal macronutrients, carbohydrate, fat and protein. The levels of these macronutrients were reduced to a similar degree such that the overall dietary composition remained unchanged.

As well as conventional lifestyle intervention, pharmacological therapies are increasingly used to combat obesity. Rimonabant is a cannabinoid-1 receptor blocker that induces weight loss and improves the cardiovascular risk profile, glycemia and insulin sensitivity in diabetic subjects [6]. The endocannabinoid system, consisting of cannabinoid type 1 (CB1) receptors and endogenous ligands is expressed widely, not only in the central nervous system but also in peripheral organs including visceral adipose tissue [18]. The effects of CB1 receptor blockers on weight, energy expenditure and calorific intake have been studied in detail in rodent models. In these studies CB1-receptor antagonism at the hypothalamic level resulted not only in reduced food intake but also a change in the composition of the diet [8,9,19]. In particular, Mathes et al. using a novel dessert protocol in female rats demonstrated a lowered calorific consumption with reduced intake of palatable food (sugar fat whip) following rimonabant treatment [19]. In addition, rimonabant has been shown in a rodent model to enhance lipolysis in adipose tissue leading to increased energy expenditure through oxidation of fatty acids and this effect was considered an important determinant of weight loss independent of food intake [7].

Inferences from these animal studies include the contention that the endocannabinoid system is an important modulator of the rewarding properties of foods by acting through specific mesolimbic areas in the brain and that CB1 receptor antagonists may have the potential in humans to reduce the intake of hedonistic type foods in favour of less energy-dense and healthier alternatives [4,20]. To date these observations remain unproven in human studies and indeed, results from our present study are at variance with these animal studies in that our patients’ dietary composition following rimonabant therapy was unchanged, with no evidence of a shift towards a healthier, less palatable diet. Instead, individuals appeared to simply reduce calorie intake by globally reducing intake of most macronutrients and several micronutrients in addition. ‘Surrogate markers’ of a healthier diet such as an increased intake of potassium or dietary fibre were not observed and in fact levels of both these nutrients fell following rimonabant therapy.

In the present study we observed a strong relationship between carbohydrate intake and blood glucose levels, with reduction in carbohydrate intake during the study being closely correlated with reduction in glycated haemoglobin independent of body weight change or total energy intake. In contrast, changes in body weight appeared to be more closely related to fat intake rather than carbohydrate intake. These data are in keeping with the growing body of evidence in favour of carbohydrate restriction as a key intervention to optimise glucose control in individuals with type 2 diabetes [10,13-15].

Micronutrient intake was also assessed in the present study. Of note, levels of several micronutrients fell following dietary and rimonabant therapy, paralleling the changes observed in macronutrients. Indeed, there appeared to be a trend for an increasing number of subjects failing to meet the Reference Nutrient Intake (RNI) for several important micronutrients following the intervention. This observation appears to suggest that some patients could be at risk of adopting a poorer quality diet following this type of dietary intervention and is an area of major concern.

### Study limitations and recommendations

This study was intended to mimic that of a clinical situation as much as possible to give an insight into how CB1 receptor antagonists impact patient’s diets and to ascertain whether further research on CB1 receptor antagonists and indeed other anti-obesity medication on dietary composition is worthwhile. There are certainly limitations to this study, namely the relatively small sample size, open design and lack of a control group. Ideally a randomised, placebo-controlled trial would have been the preferred study design but this was not feasible within the resources available to us. Another alternative would have been to study a parallel group of similar patients who lost weight through dietary intervention alone, without anti-obesity medication. This latter type of study design would have significantly enhanced the quality of this study and indeed was originally intended. Unfortunately, it was not possible to achieve meaningful weight loss through our dietary intervention alone in our intended control group of type 2 diabetic patients. Since no valid comparisons in terms of dietary changes between rimonabant-treated patients and controls are possible without comparable weight loss achieved in each group, it was decided that the rimonabant-treated patients alone would be included in the final analysis.

Detailed dietary analysis can be a difficult method to employ in clinical research but it is hoped that future research including larger controlled trials will be possible to examine dietary changes induced by both centrally acting anti-obesity drugs and other potential anti-obesity agents such as glucagon-like-peptide-1 (GLP-1) agonists. This is certainly an area worthy of further study given the potential widespread application of such agents and the knowledge gleaned from the present study suggesting significant change in dietary composition associated with CB-1 receptor antagonism. In particular, our observations that levels of some important micronutrients may fall below recommended levels of intake following anti-obesity medication is an area of special concern. We would suggest that detailed dietetic input is warranted in this situation to ensure that dietary intake of essential micronutrients is not compromised during this type of weight loss intervention.

## Conclusions

Weight loss achieved following dietary intervention in combination with rimonabant therapy occurred because of reduced energy intake. This was associated with an unselective reduction in intake of all the major macronutrients without any change to the overall composition of the diet. Thus, although pre-clinical studies support the hypothesis that CB1 receptor antagonists modulate feeding behaviour by altering the perception of palatability, our present study does not support this hypothesis in human subjects with type 2 diabetes.

## Competing interests

CH and SB declare that they have no competing interests. JS has received honoraria for lecturing and an unrestricted educational grant from Sanofi Aventis.

## Authors’ contributions

CH provided dietary input to patients in the study, performed the dietary analysis and contributed to the writing of the manuscript. SB contributed to the planning of the study and was responsible for the day-to-day running of the study. JS was principal investigator, responsible for study design and wrote the manuscript. All authors read and approved the final manuscript.

## Source of funding

Unrestricted educational grant from Sanofi Aventis.

## Role of authors

Design of study (JS,SB,CH), data collection (SB,CH), data analysis (JS,CH), manuscript preparation and review (JS,CH).
